# Safety and biocompatibility of a bionic eye: Imaging, intraocular pressure, and histology data

**DOI:** 10.1016/j.dib.2021.107634

**Published:** 2021-11-26

**Authors:** Samuel C. Eggenberger, Natalie L. James, Cherry Ho, Steven S. Eamegdool, Veronika Tatarinoff, Naomi A. Craig, Barry S. Gow, Susan Wan, Christopher W.D. Dodds, Donna La Hood, Aaron Gilmour, Shannon L. Donahoe, Mark Krockenberger, Krishna Tumuluri, Melville J. da Cruz, John R. Grigg, Peter McCluskey, Nigel H. Lovell, Michele C. Madigan, Adrian T. Fung, Gregg J. Suaning

**Affiliations:** a School of Biomedical Engineering, Faculty of Engineering, University of Sydney, Sydney, Australia; b Graduate School of Biomedical Engineering, University of New South Wales (UNSW), Sydney, Australia; c Save Sight Institute, The University of Sydney, Specialty of Clinical Ophthalmology and Eye Health, Faculty of Medicine and Health; d The Westmead Institute for medical research, Westmead, Australia; e Brien Holden Vision Institute, Sydney, Australia; f School of Optometry and Vision Science, University of New South Wales (UNSW), Sydney, Australia; g Veterinary Pathology Diagnostic Services, Sydney School of Veterinary Science, University of Sydney, Sydney, Australia; h Westmead Clinical School, Specialty of Clinical Ophthalmology and Eye Health, Faculty of Medicine and Health, University of Sydney, Sydney, Australia; i Department of Ophthalmology, Faculty of Medicine and Health Sciences, Macquarie University, Sydney, Australia; j Department of Otolaryngology, Westmead Hospital, University of Sydney, Sydney, Australia; k Faculty of Medicine and Health, University of Sydney, Sydney, Australia

**Keywords:** Visual prosthesis, Intraocular pressure, Opthalmoscopy, Biocompatibility, Histopathology, Retina

## Abstract

The data presented here are related and supplementary data to the research article “Implantation and long-term assessment of the stability and biocompatibility of a novel 98 channel suprachoroidal visual prosthesis in sheep” [1]. In Eggenberger et al., nine sheep of the Suffolk (N=2) and Dorper (N=7) breeds were implanted in the left eye with an electrically inactive, suprachoroidal retinal stimulator (Bionic Eye) for durations of up to 100 days. The surgical safety, implant stability and device biocompatibility were assessed. Intraocular pressure measurements, indirect and infrared ophthalmoscopy and optical coherence tomography were performed at fixed time points to evaluate the clinical effects of the surgery and device implantation. Post-mortem eye tissue collection and histology was performed to measure the effects of the intervention at the cellular level. The data, including a comprehensive collection of fundus, infrared, optical coherence tomography and histology images can be used as a reference for comparison with other research, for example, active retinal stimulators. Furthermore, these data can be used to evaluate the suitability of the sheep model, in particular Dorper sheep, for future research.

## Specifications Table

**Table:** 

Subject	Biomedical Engineering
Specific subject area	Intraocular pressure, clinical imaging, and histology: long-term effects of retinal implants in sheep
Type of data	Table Image Graph Figure
How data were acquired	Intraocular pressure: TonoVet rebound tonometer, Icare Oy, Helsinki, Finland, GraphPad Prism version 8.4.3 for Windows (GraphPad Software, San Diego, California USA) Indirect ophthalmoscopy: Super Quad 160 lens (Volk, Mentor OH, USA), video camera (Galaxy S3, Samsung, Seoul, South-Korea or XR500, Sony, Tokyo, Japan), OPMI operating microscope (Zeiss, Oberkochen, Germany), VLC Media Player software (Free Software Foundation, Inc., Boston, USA) Infrared fundus imaging: Wangiscope (Custom device as described in [2]), VLC Media Player software (Free Software Foundation, Inc., Boston, USA) Optical coherence tomography: Envisu 2300 system (BiOptigen, Morrisvile NC, USA) Histology: Aperio Versa 1.0.4, Leica Biosystems, Nussloch, Germany, ImageScope™ version 12.4.0.5043 (Leica Biosystems, Nussloch, Germany) Fluorescence immunohistochemistry: LSM 700 Meta Confocal microscope system and ZEN Blue software, Carl Zeiss, Germany. Iba1 immunoperoxidase and quantitative analysis: Aperio Versa 1.0.4, Leica Biosystems, Nussloch, Germany, ImageScope™ version 12.4.0.5043 (Leica Biosystems, Nussloch, Germany)
Data format	Raw Analysed Filtered
Parameters for data collection	Intraocular pressure was measured in awake sheep. Ophthalmoscopy and optical coherence tomography were performed in anaesthetised animals. Histology and immunohistochemistry was performed after fixing and paraffin embedding the tissues.
Description of data collection	Nine sheep were implanted with a suprachoroidal visual prosthesis comprising of a platinum-silicone electrode array. The animals were implanted for durations of between two days and three months.
Data source location	Institution: Graduate School of Biomedical Engineering, University of New South Wales City/Town/Region: Sydney, New South Wales Country: Australia
Data accessibility	Repository name: Mendeley Data Data identification number: Intraocular pressure, ophthalmoscopy, optical coherence tomography: DOI 10.17632/9hnz6c99py.2 Histology: DOI 10.17632/8rzc45vp77.2 Direct URL to data: Intraocular pressure, ophthalmoscopy, optical coherence tomography: http://dx.doi.org/10.17632/9hnz6c99py.2 Histology and immunohistochemistry: http://dx.doi.org/10.17632/8rzc45vp77.2
Related research article	Samuel C. Eggenberger, Natalie L. James, Cherry Ho, Steven S. Eamegdool, Veronika Tatarinoff, Naomi A. Craig, Barry S. Gow, Susan Wan, Christopher W.D. Dodds, Donna La Hood, Aaron Gilmour, Shannon L. Donahoe, Mark Krockenberger, Krishna Tumuluri, Melville J. da Cruz, John R. Grigg, Peter McCluskey, Nigel H. Lovell, Michele C. Madigan, Adrian T. Fung, Gregg J. Suaning Implantation and long-term assessment of the stability and biocompatibility of a novel 98 channel suprachoroidal visual prosthesis in sheep Biomaterials, Volume 279, 2021, 121191, ISSN 0142-9612 https://doi.org/10.1016/j.biomaterials.2021.121191. [1]

## Value of the Data

• The sheep (*ovis aries*) is a promising model for eye surgery and visual neuroscience, due to the similarities in size and shape with the human ocular anatomy [3]. The extensive dataset can be used as a reference for research involving retinal and suprachoroidal devices or to evaluate the adequacy of the ovine model for future research, therefore helping to reduce the numbers of animals used for research purposes.
• Researchers developing and studying retinal implants, in particular devices positioned in the suprachoroidal space can benefit from the data by using it as reference. Other researchers in the field of ophthalmology can use the data to evaluate the adequacy of the ovine model for their research.
• The dataset establishes a reference to which research involving surgical interventions on the eye and/or implantation of devices can be compared.

## 1 Data Description

**Note:** Animals were identified according to the experimental group to which they were assigned (duration of experiment 2 days, 1 month, 2 months and 3 months). For instance, 2D#1 was the first animal in the group implanted for a duration of 2 Days. 3M#3 was the third animal in the group implanted for 3 Months. 2D#1 and 1M#1 were of the Suffolk Breed. All other animals were of the Dorper breed.

### 1.1 Intraocular pressure (IOP)

**Fig. 1:**
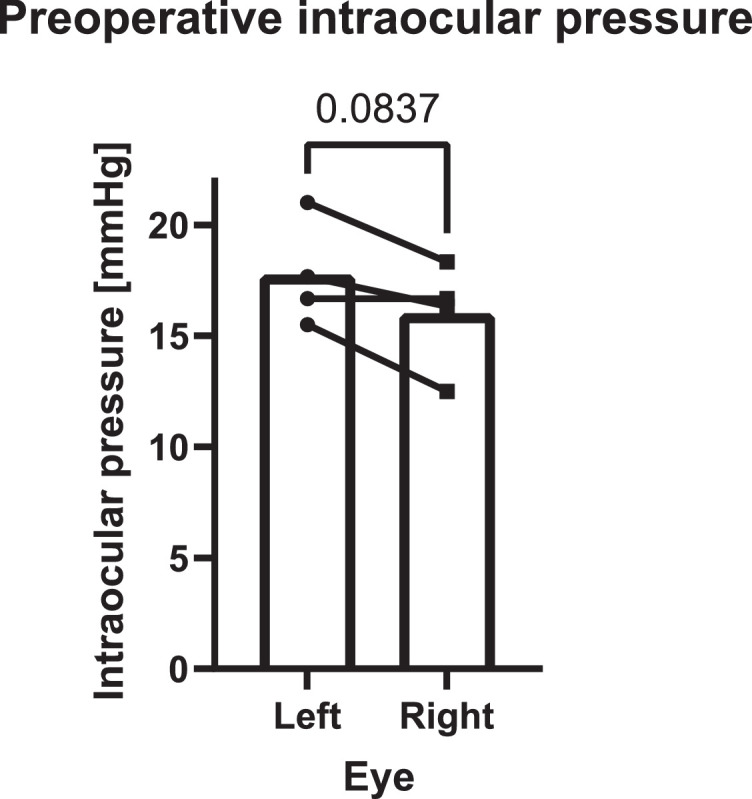
Left and right eyes baseline (preoperative) intraocular pressure for four sheep. There was no significant difference between the left and right eyes (paired Student's t-test, P=0.0837).

Fig. 1 shows the preoperative intraocular pressure (IOP) values for both eyes of four sheep. For each eye, multiple consecutive measurements were averaged together. A paired Student's t-test showed no statistical difference between the left and right eyes (P=0.0837). The average preoperative IOP in both eyes for this cohort was 16.8 ± 2.4 mmHg (mean ± SD), which is similar to reported values (16.36 ± 2.19 mmHg) [4].

**Fig. 2:**
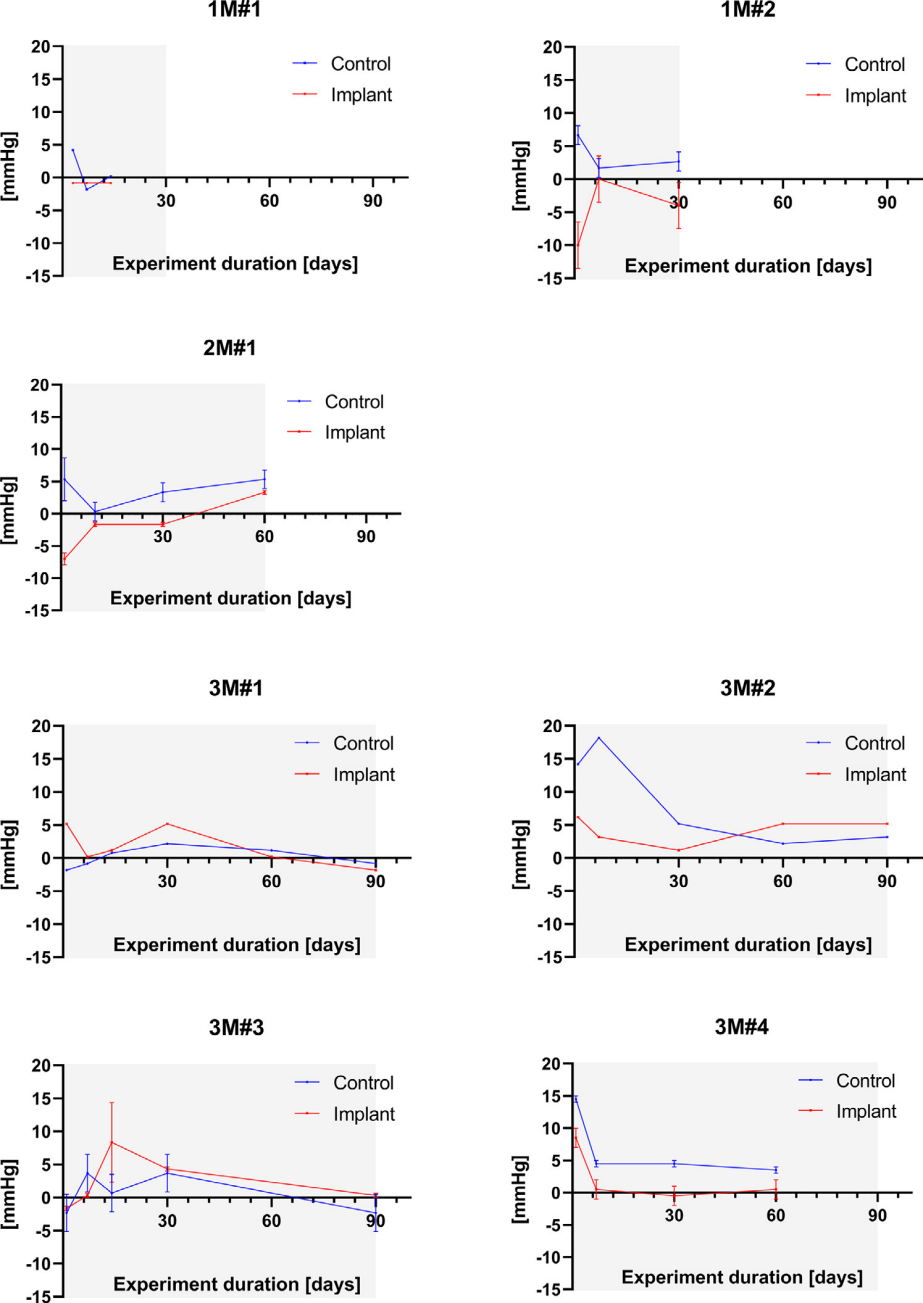
Changes in IOP compared to baseline for seven animals. For 1M#1, 3M#1 and 3M #2, where no baseline value was available, the average baseline value from all other eyes in the study (16.8 mmHg) was used. Grey areas represent the experiment duration, allowing all plots to be displayed with the same horizontal axis. Error bars represent the standard error of the mean (SEM) where multiple values were obtained on the same day.

Fig. 2 shows the changes in IOP in both eyes for seven animals implanted in the left eye for two days, one month, two months or three months respectively. When available, the preoperative baseline for each eye was subtracted from the post-operative values. In three animals (1M#1, 3M#1, and 3M #2), no baseline was available. In these cases, the preoperative baseline of the other eight (right and left) eyes were averaged together and used as baseline substitute (16.8 mmHg). Variations to baseline were plotted against time over the duration of the trials.

The data shows that postoperative variations occurred in both eyes after surgery, mostly within the first seven days after surgery. The average day 1 absolute variation from baseline was 6.4 ± 2.9 mmHg (mean ± SD) for the operated eye and 7.6 ± 5.5 mmHg for the contralateral eye. Three operated eyes increased, two decreased and one remained stable (pressure difference ≤ 2 mmHg) while five and one non-operated eyes increased and decreased, respectively.

The whole dataset is available as supplementary data in [5].

### 1.2 Ophthalmoscopy – colour and infrared fundus images (raw and aligned data)

Colour, fundus images for nine sheep of the Dorper breed are presented in [5]. Images present the appearance of the retinas and blood vessels before and after a silicone-platinum electrode array was positioned into the suprachoroidal space, in individual eyes. The rotated and cropped image stacks provide direct comparison between time points. The data provides an overview of the surgery-related changes to the retinal appearance, including transient, localized reflectivity changes at the edges of the array allowing for identification of its position.

Postoperative infrared images obtained from the same cohort and presented in [5] provide further information on the location of the electrode array in the suprachoroidal space. Using blood vessels as landmarks, the device position can be tracked over time in each animal. The same landmarks can be used to combine images from multiple imaging modalities, such as indirect ophthalmoscopy. Thus, permitting electrode array position visualisation in images where this information is missing.

### 1.3 Optical coherence tomography (OCT)

Preoperative and postoperative optical coherence tomography (OCT) rasters for four sheep of the Dorper breed are presented [5]. These images show the retinal layers and compare the retinal anatomy before and after device positioning in the suprachoroidal space. The different scales between the scan depth and scan position accentuate the stepped appearance of the retina at the array edges. In some images, the individual 600 µm platinum electrodes of the array are visible in the suprachoroidal space. Data is unavailable for 3M#3 at two and three months, and for 3M#4 at three months due to technical difficulties. All images are available as supplementary material in [5].

### 1.4 Histology – raw images

Hematoxylin and eosin (H&E) stained slides of the sheep retina, choroid, and sclera are presented here. Five eyes had an electrode array surgically implanted in the suprachoroidal space for one (N=1), two (N=2), and three months (N=3). Control scans of the contralateral eyes are also presented (N=3). The microscopic images show the retinal layers, choroid and sclera as well as the host response to the foreign body (fibrosis and inflammation). The devices were removed prior to embedding and sectioning and the pocket vacated by the electrode array is clearly visible for all implant durations. The collection of micrographs allows comparison of the effects of the intervention and presence of the device between animals and implant durations, and between control sheep retinas obtained from the same animals.

The data shows segments of detached retina. Hypertrophied retinal pigmented epithelium (RPE) can be used to differentiate *in vivo* detachments from artefactual detachments secondary to tissue processing. When present, device and/or surgery-related thinning and remodelling of the retina is visible in the scanned slides. The data shows the host response to the surgery and the silicone-platinum electrode array.

The micrographs are available as supplementary material in [6].

### 1.5 Histology - retinal thickness

**Fig. 3:**
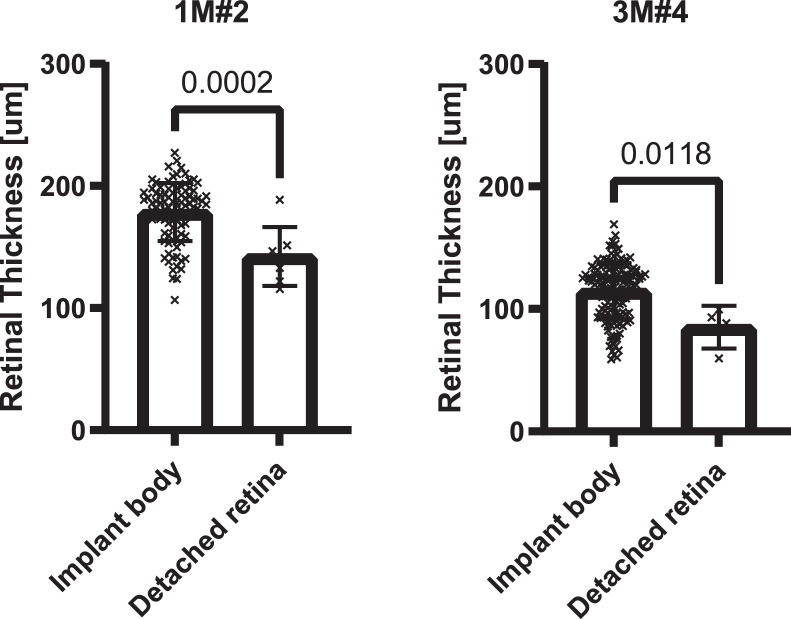
Retinal thickness over segments of detached retina. Scatter plots with bar showing mean and standard deviation for the implanted eye of two animals. Significant thinning of the detached retinal segments was observed in both cases (P=0.0002 and P=0.0118).

Fig. 3 shows the measured total thickness of the sheep retina in two animals in which non-artefactual detachments were visible. In both cases, the detached retinal segments were significantly thinner (than the retina located above the implant body; two-tailed Student's t-test, P=0.002 and P=0.0118 respectively). Table 1 summarizes the average thicknesses and standard deviations for the two sheep.

**Table 1:** Mean retinal thickness values and standard deviation for detached segments and over the body of the implant.

	Retinal thickness			
	Implant body		Detached segments	
Animal ID	Mean	SD	Mean	SD
**1M#2**	178.6	23.82	142.3	24.11
**3M#4**	114.5	22.83	85.15	17.6

**Fig. 4:**
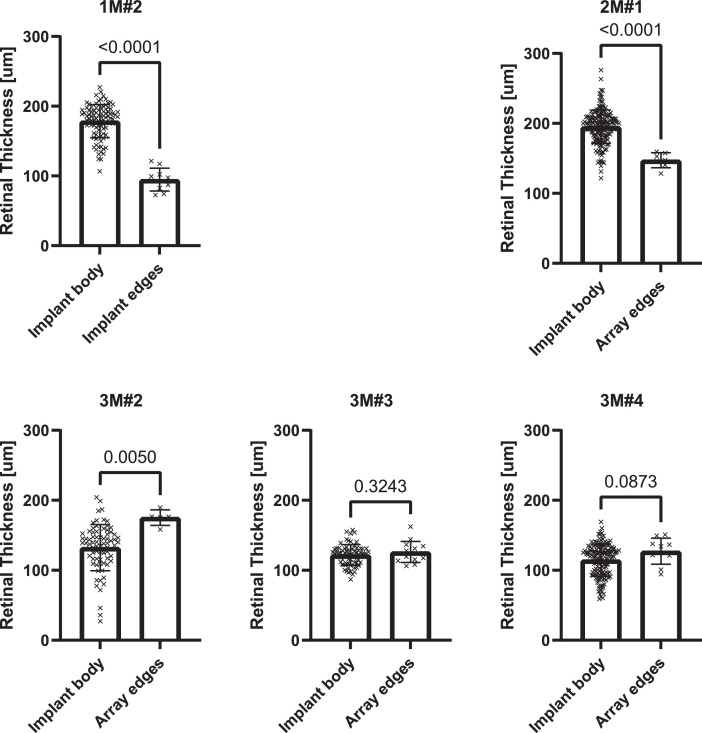
Comparison of the retinal thickness measured over the implant body and the thickness measured adjacent to the edges of the electrode arrays. The difference was significant in 1M#2, 2M#1, and 3M#2.

Fig. 4 compares the retinal thickness measured over the implant body and the thickness measured adjacent to the edges of the electrode arrays. The difference was not significant in 3M#3 and 3M#4, P=0.3243 and P=0.0873, respectively). The retinas were significantly thinner adjacent to the device edges in three cases (3/5). Average retinal thicknesses, standard deviations and p-values are summarized in Table 2.

**Table 2:** Retinal thickness values over the body of the implant and at the implant edges. P-values showing significant thinning in the retina close to the implant edges and calculated using an unpaired Student's t-test are shown.

Animal ID	Retinal thickness [µm]				P
	Implant body		Implant edges		
	Mean	SD	Mean	SD	
**1M#2**	178.6	23.82	94.73	16.42	<0.0001
**2M#1**	195.2	23.86	147.4	10.68	<0.0001
**3M#2**	132.3	32.99	175.3	11.05	0.005
**3M#3**	121.8	14.77	126.1	15.09	0.3243
**3M#4**	114.5	22.83	127.2	18.66	0.0873

Samples were collected at variable distances from the *area centralis*, sometimes at the transition from visual to non-visual retina. Raw data is available as supplementary material in [6].

### 1.6 Immunohistochemistry

The data present a collection of fluorescence micrographs obtained from sheep implanted for zero (control), one, two and three months with a suprachoroidal visual prosthesis. A combination of GFAP and LM-opsin immunostaining allow for the assessment of Muller glia activation and effects on the photoreceptor layer. Iba1 immunostaining allows for visualisation of the macrophage/microglia cells.

All fluorescence micrographs were imaged using confocal microscopy and are available as supplementary data in [6].

The data also present a collection of Iba1 immunoperoxidase micrographs for three control eyes and three eyes implanted for three months, as well as their corresponding negative controls. Quantitative analysis was performed using the Aperio ImageScope “Positive Pixel Count” algorithm on the retinas [7, 8]. The regions of interest were manually defined around the retinas. All micrographs and algorithm outputs are available as supplementary data in [6], including but not limited to the ‘Positivity’ (ratio of the number of Iba1-positive pixels to the total number of negative and positive pixels in the region of interest).

## 2 Experimental Design, Materials and Methods

### 2.1 Intraocular pressure

Preoperative intraocular pressure (IOP) was measured in four sheep (ovis aries) of the Dorper breed. Postoperative IOP measurements were obtained in seven animals (Suffolk breed N=1, Dorper breed N=6) on day one,

Intraocular pressure (IOP) measurements were made using a TonoVet rebound tonometer (Icare Oy, Helsinki, Finland), in the holding pen and without anaesthesia. Preoperative IOP was measured after the acclimatisation period in the animal facilities (Graduate School of Biomedical Engineering (GSBmE), UNSW Sydney, Australia) and at least two days prior to surgery. Individual baseline IOP was calculated, for each eye, as the mean of two readings for one animal or three measurements for a further three sheep. IOP values were obtained on days 1 and 7 post-surgery, then on days 30, 60 and 90, where applicable. One IOP value was obtained at day 3 instead of day 1 after surgery. One measurement was performed on day 10 instead of 7 because of a secondary surgical intervention. Similarly, no IOP values were obtained from 3M#3 at the two-month mark because of a secondary surgery. The 30-, and 90-day IOP measurements were not available on the last experimental day for 1M#1 and 3M#4, respectively. Additional measurements at 14 days were made in a subset of the cohort (N=3). Whenever possible, six successive readings were obtained, according to the device's mode of operation. The mean of consecutive measurements was used whenever sufficient consecutive values could not be obtained.

IOP values were recorded in GraphPad Prism version 8.4.3 for Windows (GraphPad Software, San Diego, California USA). Whenever multiple preoperative measurements were available for a single eye, these were averaged to obtain the IOP baseline for that eye. An unpaired, two-tailed Student's t-test was performed in GraphPad to compare the average IOP in the left and right eyes. Individual baselines were averaged together to serve as reference for the other eyes. IOP baseline values were subtracted from the postoperative measurements and plotted versus time.

### 2.2 Ophthalmoscopy – colour and infrared fundus images (raw and aligned data)

Indirect ophthalmoscopy was used to obtain colour fundus images before and after implantation of a suprachoroidal visual prosthesis in anaesthetised animals. The data was obtained as video files using a Super Quad 160 lens (Volk, Mentor OH, USA) and video camera (Galaxy S3, Samsung, Seoul, South-Korea or XR500, Sony, Tokyo, Japan), passed through the optics of an OPMI operating microscope (Zeiss, Oberkochen, Germany). Infrared fundus imaging was performed postoperatively using a technique described in [2] and recorded as video files.

All video files were visualised frame by frame after deinterlacing (automatic) using VLC Media Player software (Free Software Foundation, Inc., Boston, USA). Visibility of the region of interest (whenever possible, centred around the location of the electrode array) and image focus were used as criteria to select the most representative frames. For each imaging technique and each eye (indirect ophthalmoscopy or infrared imaging), blood vessels visible in all images were identified and used as landmarks to rotate and scale the data in Fiji [9, 10] and using the “align image by line ROI” plug-in [11]. The resulting stacks were then cropped to the region of interest.

Both techniques were applied to obtain images directly after surgery and then monthly until the animal was sacrificed (up to three months). Additionally, preoperative colour fundus images were acquired as baseline.

### 2.3 Optical coherence tomography (OCT)

OCT imaging was performed in anaesthetised animals with an Envisu 2300 system (BiOptigen, Morrisvile NC, USA). Images were obtained from four sheep of the Dorper Breed before device implantation, directly postoperatively and then at the one-, two and three-month mark.

### 2.4 Histology and retinal thickness

Tissue was fixed and processed according to the methods described in the co-publication [1]. Micrographs were digitised using an Aperio Versa 1.0.4 (Leica Biosystems, Nussloch, Germany) slide scanner and viewed in ImageScope™ version 12.4.0.5043 (Leica Biosystems, Nussloch, Germany). All retinal detachments were identified visually. Artefactual detachments, characterised by non-hypertrophied retinal pigmented epithelium, were excluded. Retinal thickness measurements were performed by placing markers every 200 µm along the retinal pigmented epithelium and then measuring the thickness perpendicular to the retina with the ruler tool. The location of the implant edges was identified visually (location of the pocket left by the electrode array, which was removed prior to embedding and sectioning). Tissue located 600 µm on either side of end the pocket was categorized as “adjacent to the device edge”. In instances where the retinal layers were separated due to processing artefacts, the ruler tool was used to measure each segment separately. The corresponding sub-layer thicknesses were then added to obtain the local total retinal thickness. Values were compiled in MS Excel (Microsoft Corporation, Redmond, Washington, USA) and analysed in GraphPad Prism version 8.4.3 for Windows (GraphPad Software, San Diego, California USA). When multiple slides were available for a single eye, all thickness values from the slides were combined into one dataset prior to analysis.

Mean thickness and standard deviation were calculated for each eye and each type of segment (body of the implant, implant edges and detached retina). Two-tailed, unpaired Student's t-tests were performed for each eye (where data was available) to compare the mean thickness over the implant body and one of the regions of interest (implant edge or detached retina). The test's assumptions (approximately normal distributions of the continuous dependent variables, the absence of outliers and independence between the observations) were considered fulfilled despite the low number of samples obtained from the regions of interests (due to their small size).

### 2.5 Fluorescence immunohistochemistry

Tissues were fixed and processed, and paraffin sections cut, according to the methods described in the co-publication [1]. Briefly, sections were incubated with primary antibodies: GFAP-Cy3 conjugate (mouse IgG; 1:800); opsin-red/green (opsin R/G) (rabbit IgG; 1:500, 1:1000) (Merck Millipore) and Iba1 (goat IgG; 1:200) (Abcam, USA). Immune complexes were detected with secondary antibodies to goat IgG, mouse IgG, or rabbit IgG, conjugated with Alexa-Fluor 488, 555, or 647 (Invitrogen, USA). Nuclei were counterstained with Hoechst 33258 (1:10,000) prior to mounting in glycerol:PBS (80:20). Representative regions of the slides were captured using an LSM 700 Meta Confocal microscope system and ZEN Blue software (Carl Zeiss, Germany). The images were then assembled in Photoshop CS software (Adobe Corporation, USA).

### 2.6 Iba1 immunoperoxidase and quantitative analysis

Tissues were fixed and processed, and paraffin sections cut, according to the methods described in the co-publication [1]. Briefly, 4um sections were heat antigen retrieved in high pH buffer (Target Retrieval Solution, S2367, DAKO®). The following steps were performed with DAKO® Link Autostainer: blocking (Bloxall, SP6000, VectorLabs®), primary antibody (macrophage/microglia, clone Iba1, BioCare Medical®, rabbit polyclonal, 1:200), secondary (Envision K5007, DAKO®), chromogen ImmPACT Nova Red (SK4805, Vector Labs®), counterstained in haematoxylin (Whitlocks, VPDS Histo Lab Manual©, 2021), cleared and mounted. Primary antibody was omitted in the negative controls. Sections were scanned (40x magnification) with Aperio Versa (Leica Biosystems®, UK).

Retinas were manually outlined using the Pen Tool in ImageScope™. The retinal pigmented epithelium was carefully avoided to prevent melanin-related false positives. The following algorithm parameters were used: hue value = 0, hue width = 0.4, colour saturation threshold = 0.25, intensity threshold of weak positive pixels upper limit = 160, intensity threshold of positive pixels upper limit = 140, intensity threshold of strong positive pixels upper limit = 120, intensity threshold of strong positive pixels lower limit = 0, intensity threshold of negative pixels upper limit = -1.

## Declaration of Competing Interest

The authors declare the following financial interests/personal relationships which may be considered as potential competing interests: Gregg Suaning reports financial support was provided by NH&MRC. Gregg Suaning has patent issued to New South Innovations.
